# The hierarchical response of human corneal collagen to load

**DOI:** 10.1016/j.actbio.2017.11.015

**Published:** 2018-01

**Authors:** J.S. Bell, S. Hayes, C. Whitford, J. Sanchez-Weatherby, O. Shebanova, C. Vergari, C.P. Winlove, N Terrill, T. Sorensen, A. Elsheikh, K.M. Meek

**Affiliations:** a School of Optometry and Vision Sciences, Cardiff University, Maindy Road, Cathays, Cardiff CF24 4HQ, UK; b Cardiff Institute of Tissue Engineering and Repair, Cardiff University, 10 Museum Place, Cardiff CF10 3BG, UK; c School of Engineering, University of Liverpool, The Quadrangle, Brownlow Hill, Liverpool L69 3GH, UK; d Diamond Light Source Ltd, Diamond House, Harwell Science & Innovation Campus, Didcot, Oxfordshire OX11 0DE, UK; e Department of Physics and Astronomy, University of Exeter, Physics Building, Stocker Road, Exeter EX4 4QL, UK; f NIHR Biomedical Research Centre for Ophthalmology, Moorfields Eye Hospital NHS Foundation Trust and UCL Institute of Ophthalmology, UK

**Keywords:** Collagen, Cornea, X-ray scattering, Biomechanics, Microstructure

## Abstract

Fibrillar collagen in the human cornea is integral to its function as a transparent lens of precise curvature, and its arrangement is now well-characterised in the literature. While there has been considerable effort to incorporate fibrillar architecture into mechanical models of the cornea, the mechanical response of corneal collagen to small applied loads is not well understood. In this study the fibrillar and molecular response to tensile load was quantified using small and wide angle X-ray scattering (SAXS/WAXS), and digital image correlation (DIC) photography was used to calculate the local strain field that gave rise to the hierarchical changes. A molecular scattering model was used to calculate the tropocollagen tilt relative to the fibril axis and changes associated with applied strain. Changes were measured in the D-period, molecular tilt and the orientation and spacing of the fibrillar and molecular networks. These measurements were summarised into hierarchical deformation mechanisms, which were found to contribute at varying strains. The change in molecular tilt is indicative of a sub-fibrillar “spring-like” deformation mechanism, which was found to account for most of the applied strain under physiological and near-physiological loads. This deformation mechanism may play an important functional role in tissues rich in fibrils of high helical tilt, such as skin and cartilage.

**Statement of Significance:**

Collagen is the primary mediator of soft tissue biomechanics, and variations in its hierarchical structure convey the varying amounts of structural support necessary for organs to function normally. Here we have examined the structural response of corneal collagen to tensile load using X-rays to probe hierarchies ranging from molecular to fibrillar. We found a previously unreported deformation mechanism whereby molecules, which are helically arranged relative to the axis of their fibril, change in tilt akin to the manner in which a spring stretches. This “spring-like” mechanism accounts for a significant portion of the applied deformation at low strains (<3%). These findings will inform the future design of collagen-based artificial corneas being developed to address world-wide shortages of corneal donor tissue.

## Graphical abstract

**Figure:**
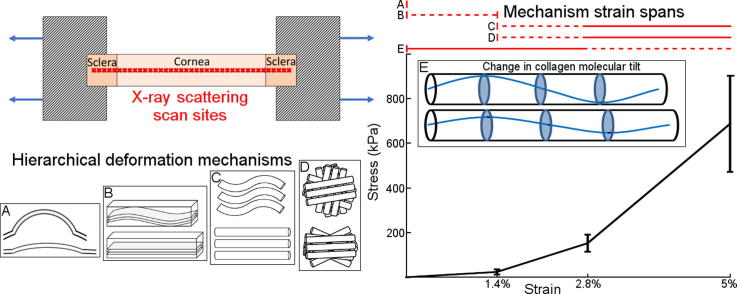

## 1 Introduction

Collagen is the most abundant structural protein in the mammalian body, and plays a primarily mechanical role in facilitating normal organ processes, whilst conveying structural integrity and resistance to damage and injury. The mechanical environments of each part of the body differ enormously, and it is through variations in the hierarchical structure of collagen and its interactions with cells and the extracellular matrix that these environments are maintained. The mechanical properties of collagen are of great interest, as many degenerative diseases and surgical procedures perturb the collagen network, leading to improper function.

The type I tropocollagen molecule is the basic building block of most collagenous tissues, and consists of three polypeptide chains arranged in a superhelix measuring approximately 280 nm long and 1.5 nm in diameter. It is very stiff, with tensile modulus ranging from 2.9 GPa to in excess of 9 GPa depending upon species and tissue type [1]. Tropocollagen molecules are arranged in a staggered array with a highly ordered offset of 64–67 nm, referred to as the D-period [2]. Arrays of molecules form fibrils, which can vary in diameter from 12 nm to over 500 nm [3]. An intermediate structure of microfibrils exists in some tissues, with a supertwisted morphology comprising 5 molecules, with molecules belonging to one microfibril interdigitating with its neighbours [4]. The extent to which the molecules are tilted with respect to the fibril axis has been suggested to vary between tissues, with measurements of approximately 5° in large heterogeneous fibrils (typical of skeletal tissues such as tendon), and up to 17° in the thinner, uniform sized corneal fibrils [5], [6] At larger hierarchical scales the stiffness of collagenous structures reduces, due to an increasing number of deformation mechanisms [7]. The primary deformation mechanism is dependent on the geometry of the tissue: for highly aligned tissues such as tendon and ligament, the relative sliding of fibrils dominates [8], while in tissues with more complex patterns of collagen arrangement such as cartilage and blood vessels, reorientation of the fibrillar architecture also plays a fundamental role [9], [10].

**Fig. 1:**
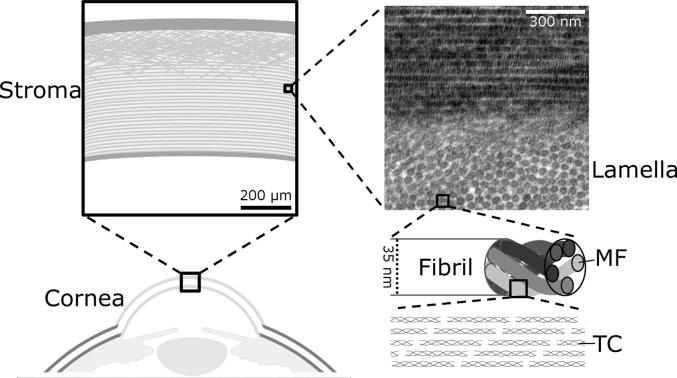
Hierarchical arrangement of collagen in the cornea. Subfibrillar illustrations are not to scale – see [4] for an atomistic interpretation. Abbreviations: MF – microfibril; TC – tropocollagen molecule. (Transmission electron microscopy image courtesy of Nada Aldahlawi, unpublished work).

The hierarchical deformation behaviour of collagen is a topic of significant current interest, and recent pioneering work has measured the mechanisms by which collagen confers mechanical toughness to skin [11] and tendon [8], the most dominant of which is interfibrillar slippage. At a molecular scale, it has been suggested that the geometry of the tropocollagen molecule permits pulsed intermolecular slippage when the elastic limit of a fibril is reached [7], providing a further source of toughness. While these characteristics are vitally important in the function of organs that undergo large deformations, there has been little study of the hierarchical deformation mechanisms in tissues that undergo small deformations, such as the cornea. The collagen network in the cornea is unique, in that it confers both mechanical stability and transparency, the latter due to the regular spacing and size of collagen fibrils, which are arranged into approximately 240 flattened and highly aligned lamellae [12], [13]. The cornea is the primary lens of the eye, and as such must maintain precisely its curvature in order to maintain clear vision. In health, the human cornea has been shown to deform little over physiological ranges of intraocular pressure (IOP) [14], [15], however degenerative diseases such as keratoconus [16] and invasive surgical procedures [17], [18], [19] perturb the collagen network, leading to changes in corneal morphology that affect vision. While the anisotropic collagen network in cornea has been used to inform mechanical models [20], [21], [22], the deformation mechanisms of corneal collagen remain largely unknown. The regular fibrillar structure of corneal collagen (Fig. 1) lends itself to X-ray scattering studies, which reveal information about the fibril spacing, diameter and orientation [23], measurements that cannot be obtained from other tissues, as well as molecular morphology and arrangement. The cornea therefore could be an ideal tissue for elucidating the fine structural response of collagenous tissues in general.

The healthy intact cornea deforms almost perfectly elastically under quasi-static pressure testing [24], [25], suggesting there is little reorientation or interfibrillar/interlamellar sliding, which require significant energy expenditure [26]. The majority of deformation due to increases in IOP occur in the periphery [27] with the stiffer central and paracentral region [28], [29] maintaining approximately the same shape. The lower stiffness of the periphery is likely associated with the transition in the arrangement of fibrils from predominantly radial to predominantly circumferential [30] meaning proportionally less collagen is recruited in the region during changes in IOP. The network of elastic fibre structures, that predominantly exists in the peripheral cornea [31], likely contributes to this elastic response, but it must be accompanied by an efficient deformation mechanism within the collagen network to minimise viscous loss and wear. This may be associated with the straightening of some hierarchical crimp [32], or of small deformations within the fibrils.

In order to elucidate the hierarchical deformation mechanisms of the collagen network, fresh human corneas were tested via quasi-static extensometry (tensile strip testing) and simultaneously imaged using small- (SAXS) or wide- (WAXS) angle X-ray scattering. Extensometry was chosen for this study as it is a simple, unambiguous test that is compatible with confined X-ray scattering stages and is easily analysed. Digital image correlation (DIC) was carried out to determine the local distribution of strain, and its relationship with local variations in the molecular and fibrillar architecture. Lastly, a scattering model was developed to quantify molecular tilt relative to the fibril axis. It is important for the reader to note that the objective of the study was not to simulate the response of the cornea to the in vivo mechanical environment but rather to determine the deformation mechanisms of corneal collagen at small, well-defined applied strains.

## 2 Materials and methods

### 2.1 Specimen preparation

In accordance with the tenets of the Declaration of Helsinki, a total of 17 post-mortem human donor cornea-scleral disks, aged between 21 and 90 years old, were obtained from UK eye banks. 9 corneas were assigned to a SAXS study and 8 to a WAXS study. The tissue was received following storage in culture media at a temperature of 37 °C for a period of 2 months. In order to reverse the tissue swelling that occurred during storage and return the corneas to near physiological hydration, the organ culture was supplemented with a 15% dextran solution for a period of 2 days prior to data collection.

Immediately prior to data collection, a 3.5 mm × 20 mm strip was cut along the vertical meridian of each cornea-scleral disk using a custom-made cutting device. The thickness of the corneal strip was recorded using an ultrasound pachymeter (DGH Pachmate 55; DGH Technologies, Exton, PA).

### 2.2 Extensometer

A custom-built extensometer comprising a piezo linear stage (Q-545-240, PI, driven by PIShift E-871 controller) and 4.9 N tension/compression load cell (Model 31, RDP) was used to apply static loads to the tensile strips. The stage and load cell were secured to fixed arms, which in turn were attached to removable serrated paddles, on to which each endmost 2 mm of the tensile specimen was carefully adhered using cyanoacrylate adhesive. Strips were cut, fastened and tested in the same way in all experiments, meaning geometrical contributions to local force distributions should be the same for all samples and not lead to results bias. The stage and load cell were mounted on a bespoke base plate that included a large central hole to allow the beam to reach the entire specimen.

The extensometry protocol used in this study is strain-controlled, rather than stress-controlled (as the cornea is in vivo). This is in order to measure the relative contribution of measurable deformation mechanisms to the applied strain. A preliminary experiment was carried out to ascertain the strains to be applied to the specimen. Synchrotron X-ray beamline time constraints limited the number of specimens and scans per specimen, so to adequately power the study 4 strain increments were applied. Increments were chosen that best covered the regions of interest on the nonlinear stress-strain curve [33], which includes features commonly known as the “toe” region, followed by a “heel” with increasing gradient, then a linear region that extends until failure. The tensile strain that gives rise to a stress equal to physiological hoop stress (10–20 kPa assuming physiological morphology [34]) was also measured. The preliminary study found the physiological stress to be reached at an average strain of 1.4%, the centre of the heel at 2.8% and the commencement of the linear region at 5%. A further strain of 8% was chosen for comparison with other extensometry studies, with higher strains rejected due to unreasonable relaxation times. A series of X-ray scattering patterns were obtained along the central axis of the specimen at each of these strain increments, and additionally at the initial tare preload, which was used to remove slack from the specimen. The tare loading protocol involved applying 50 µm longitudinal deformations to the specimen until a force was registered. Given the average sample length of 16 mm, the resulting error in zero-point strain was therefore up to 0.3%.

### 2.3 X-ray scattering

Small-angle X-ray scattering (SAXS) [17] was carried out on Beamline I22 at Diamond Light Source (Didcot, UK), using an X-ray beam of wavelength 1 Å (12.4 keV), with an approximately elliptical profile with major axis parallel to the direction of stretch. The beam profile major and minor axial lengths at the specimen were approximately 300 µm and 150 µm. A Pilatus 2P3M photodetector (Dectris, Switzerland) collected scattered light at a distance of 6.8 m from the specimen, with the majority of the scatter path in an evacuated tube to reduce air scattering. This setup allowed examination of the fibrillar architecture of corneal collagen at length scales ranging from 10 nm to 100 nm. The 58.380 Å peak associated with the [0 0 1] crystal plane reflection of powdered silver behenate was used to centre and calibrate the images.

Wide-angle X-ray scattering (WAXS) was carried out on Beamline I02 at Diamond Light Source, using an X-ray beam of wavelength 0.979 Å (1.27 keV) and an elliptical beam profile of 50 µm × 90 µm. A Pilatus 3S6M photodetector (Dectris, Switzerland) collected scattered light at a distance of 0.3 m from the specimen. This setup allowed examination of the molecular arrangement of corneal collagen at length scales ranging from 0.2 nm to 2 nm. The 3.04 Å peak associated with the [1 0 4] reflection of calcium carbonate was used to centre and calibrate the images.

**Fig. 2:**
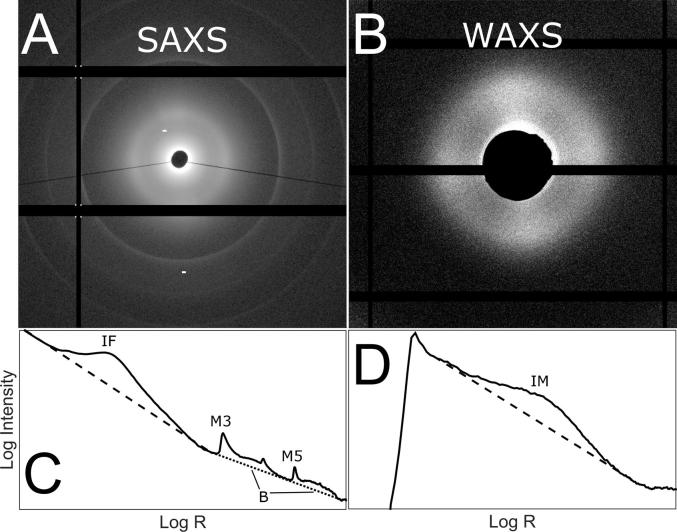
Illustrations of X-ray diffraction images and peak analysis. A. Example SAXS scatter pattern. B. Example WAXS scatter pattern. C Radial SAXS plot corresponding to panel A. D. Radial WAXS plot corresponding to panel B. Labelled peaks: Interfibrillar spacing (IF); Meridionals 3 and 5 (M3, M5); Bessel-function shaped fibril cylinder transform (used to measure fibril diameter) (B); Intermolecular spacing (IM). Dotted and dashed lines correspond to exponential background subtractions.

Example SAXS and WAXS images, and the associated radial scattering intensity profiles are shown in Fig. 2. Features of interest at the fibrillar scale were the interfibrillar spacing, fibril diameter, D-period and the in-plane (polar) arrangement of fibrillar collagen. At the molecular scale the intermolecular spacing, collagen residue spacing and polar arrangement of molecules were of interest.

**Fig. 3:**
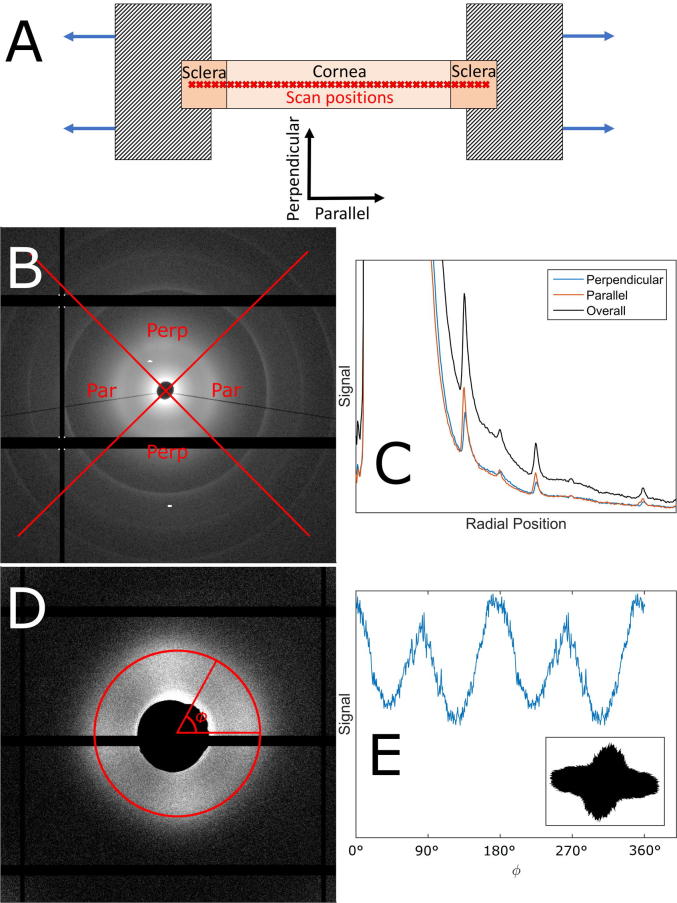
Illustrations of the experiment and analysis protocols. A. Schematic of the extensometry experiment with scan positions overlaid on the strip. B. Example SAXS pattern showing regions defined as parallel and perpendicular to the applied strain, for axial scattering (the regions are swapped for lateral scattering). C. Corresponding radial intensity plot for B, showing meridional peaks 3–8 as an example. D Example WAXS pattern with overlaid calculation for polar dependence of intermolecular peak. E. The azimuthal distribution of signal in the intermolecular peak in panel D reveals the polar distribution of molecules (after reflection-based removal of gridline). The process is the same for calculating the fibril distribution from SAXS images. Insert: Polar patch plot of the molecular distribution shown in panel E, used for compactness when showing multiple distributions across a specimen.

At each strain increment starting with the tare load, scatter patterns were obtained at 300 µm axial intervals close to the central axis of the strip, and at least 1 mm from a cut edge (Fig. 3A). Each pattern was obtained using a 1 s exposure, and between exposures the beam was blocked to minimise specimen dose. The total scan time at each strain increment was 90 s. Each strain increment was applied over 30 s, and then allowed to relax for (10, 15, 20, 30) minutes for (1.4%, 2.8%, 5%, 8%) strains. This was found to achieve approximately 80% of equilibrium stress relaxation in each case, and thereby ensuring no significant stress relaxation occurred during data collection. The specimens were sprayed with distilled water using a perfume atomizer at regular intervals to minimize drying due to evaporation. The hydration protocol was developed in a preliminary study that maintained specimen hydration in the absence of load to within a band of ±2%. Excessive drying due to evaporation typically manifested in an increase in tensile force, which relaxed rapidly when the sample was sprayed. Samples that were found to dry excessively were rejected from the study. It should be noted that load-induced volumetric changes are accompanied by changes in hydration.

### 2.4 Digital image correlation

Fiducial paint markers were sprayed onto tensile strips prior to attachment to the extensometer. Spray paint (black and white, Valspar) was found to make no discernible difference to SAXS images, and the extra WAXS peaks did not overlap with any features of interest. The paint speckles optimised the DIC process and were discrete enough not to provide another surface layer to the specimens. It has been shown in a separate study that the addition of such a speckle pattern to specimens does affect their mechanical response [25].

In order to determine strain fields, photographs of the specimen were taken immediately after each WAXS line scan, by removing the extensometer from the primary stage and placing it under a dissection microscope. Acquired images were analysed using previously described custom software for semi-automatic digital image correlation and tracking [35]. This allowed the local strain field to be calculated reliably to a resolution of approximately 300 µm. The extensometer was sufficiently rigid that moving it did not perturb the specimen.

### 2.5 Scatter analysis

All analysis was carried out using bespoke software developed in-house in the Matlab (Mathworks) environment. A detailed explanation of the analysis procedures for SAXS and WAXS of collagen can be found elsewhere [36], [37] and is described briefly here. Images were centred and converted to cylindrical polar form, with *z* representing pixel intensity, and data were split into two circumferential populations: parallel (within ±45° of applied load in the positive and negative directions), and perpendicular (Fig. 3B and C).

For WAXS, two exponential background subtractions were made to isolate the intermolecular and collagen residue spacing peaks. Gaussian fits were applied to both peaks. The angular dependence of the integrated intermolecular peak (Fig. 3D) was used to calculate polar distributions of molecular orientation (Fig. 3E). These distributions can be represented as polar patch plots (Fig. 3E, insert).

For SAXS, exponential background subtractions were made to isolate the interfibrillar peak and the Bessel peaks associated with the cylinder scattering of the fibrils (see Fig. 2C). Gaussian fits were applied to the reduced interfibrillar peak and meridionals 3, 5, 8, 9 and 12 to calculate accurately the average interfibrillar spacing and D-period, respectively. A Bessel function was fitted to calculate the average fibril diameter. The angular dependence of the integrated interfibrillar peak was used to calculate the angular distribution of fibrillar orientation at each scan position.

### 2.6 Scattering model

Polar orientation plots reflecting the angular distribution of tropocollagen molecules can be calculated readily from WAXS scatter images as discussed above. These plots are a convolution of the fibrillar orientation and the molecular tilt relative to the fibril axis (see Fig. 1). To determine changes in molecular tilt associated with strain, a scattering model was written based upon the assumption that tropocollagen molecules can be represented as helices with axis parallel to the respective fibril (an approximation of the geometry suggested in [4]). This model uses an approach described in detail in [38]. A brief summary follows.

Assuming that at each position on the fibril adjacent molecules are parallel to one-another, the scatter pattern from such a fibril can be approximated as a sum of the scatter patterns from an arrangement of straight lines at a tilt relative to the fibril axis equal to the helical tilt (illustrated in Results). To model the intermolecular peak, molecules are approximated as straight lines, which are represented as lines of closely-spaced finite spheres. Spheres are used instead of lines for mathematical simplicity. The structure factor of a sphere is

$$f(S)=\frac{2\pi \mathrm{RS}\cos(2\pi \mathrm{RS})-\sin(2\pi \mathrm{RS})}{4\pi^{2}R^{3}}$$

where *S* is the magnitude of the scattering vector and R is the radius of the sphere. The structure factor associated with an arrangement of N spheres is

$$F(S)=\overset{N}{\underset{n=1}{\sum }}f(S)e^{2\pi iS\cdot a_{n}}$$

where **a**_n_ is the position vector of the nth sphere. The scattered intensity, as measured with a detector, is

$$I(S)=F(S)F^{}(S)$$

and to construct a scatter image **S** is varied to correspond with scattering to each pixel of the image. The model comprised 97 lines of 400 spheres. Each sphere was 0.05 Å in diameter, and separated on each line by 0.1 Å. The lines were arranged in a hexagonal lattice with nearest neighbour spacing 1.9 Å, and corresponding Bragg spacing 1.71 Å [39]. Corneal WAXS patterns show only one discernible peak associated with the intermolecular spacing, indicating that the coherence length is small. To reflect this in the model, a preliminary experiment was carried out to determine the extent of disorder required to produce a single peak of correct width, and it was found that randomly displacing each line by up to 0.6 Å in *x* and *y* yielded the closest match. It should be noted that the packing arrangement of collagen molecules is still not clear [6], however for the purpose of determining molecular tilt from comparison of SAXS and WAXS patterns, the lattice arrangement had little effect. To produce a scatter image for a single fibril with a particular molecular tilt, the line model was run 180 times at 2° azimuthal increments, with lines tilted at the requisite inclination angle. The resulting image was then convoluted with fibril orientation data in central cornea from SAXS experiments to produce a modelled WAXS image. The modelled molecular orientation distributions at varying pitch were compared with those observed experimentally using a least squares fit.

### 2.7 Statistical methods

Statistics are stated as mean ± SEM. Statistical significance was calculated using a one-sided, unpaired Student’s *t*-test, with the null hypothesis rejected at the 95% confidence interval. Graphical confidence intervals are presented as mean ± SEM.

## 3 Results

### 3.1 Changes in collagen structure

**Fig. 4:**
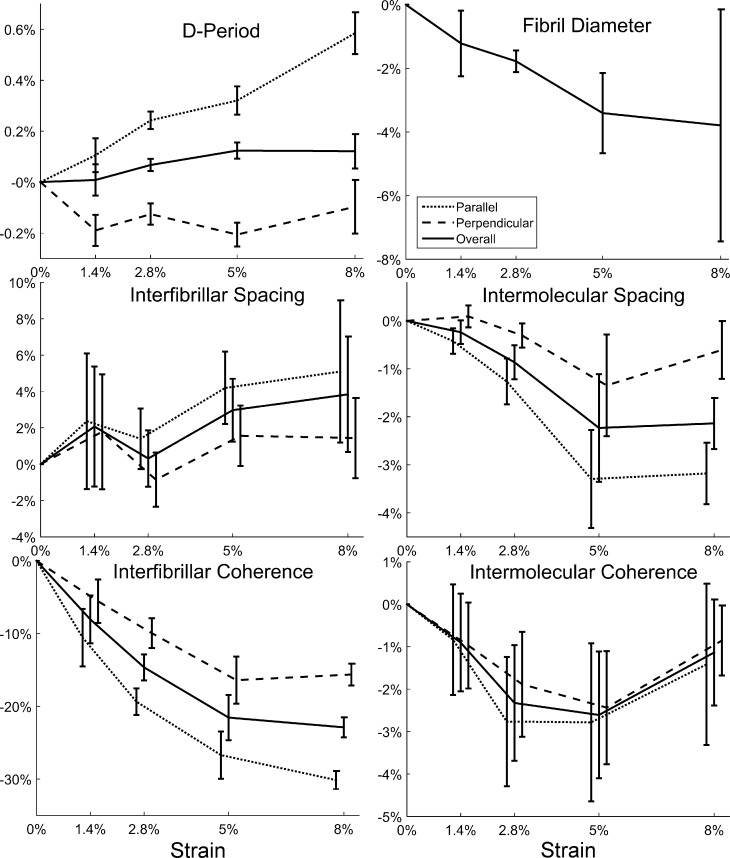
Averaged change in X-ray scattering metrics associated with strain. Error bars show SEM and are offset in *x* to avoid overlap. Parallel and perpendicular fibril diameters could not be ascertained due to loss of peak shape when strained.

A graphical overview of the X-ray scattering results taken from the lengthways central 2 mm of the specimens is shown in Fig. 4. When averaged over all fibril orientations, the D-period appeared to be unaffected by changes in strain. However, measurement of the same parameter in just the fibrils aligned parallel to the direction applied strain, revealed a significant increase in D–periodicity between 2.8% and 8% applied strain (p < .001), implying fibril elongation. In the orthogonal direction the mean D-period dropped significantly (p < .001) following the 1.4% strain increment, implying fibril shortening, and remained relatively unchanged thereafter.

Fibril diameter, measured only as an overall average due to limited signal, showed a negative trend with strain that was significant at the 2.8% and 5% increments (p < .05). As strain increased the Bessel function fit became poorer, suggesting some fibrils were increasingly variable in diameter, losing their cylindrical shape, or coming into contact with one another.

Although statistically insignificant, the spacing between fibrils appeared to increase slightly as a function of strain, whilst the inter-fibrillar coherence, which provides a measure of crystallinity or the regularity of the fibril spacing [40], [41], decreased significantly at strains of 2.8% and above (p < .001). The intermolecular spacing showed the converse, with collagen molecules aligned parallel to the direction of strain moving closer towards each other as strain increased (p < .05), but showing little change in intermolecular coherence. No change was measured in the residue spacing. It was noted in all experiments that despite minimizing drying due to evaporation the specimens became more opaque with increased tensile strain.

**Fig. 5:**
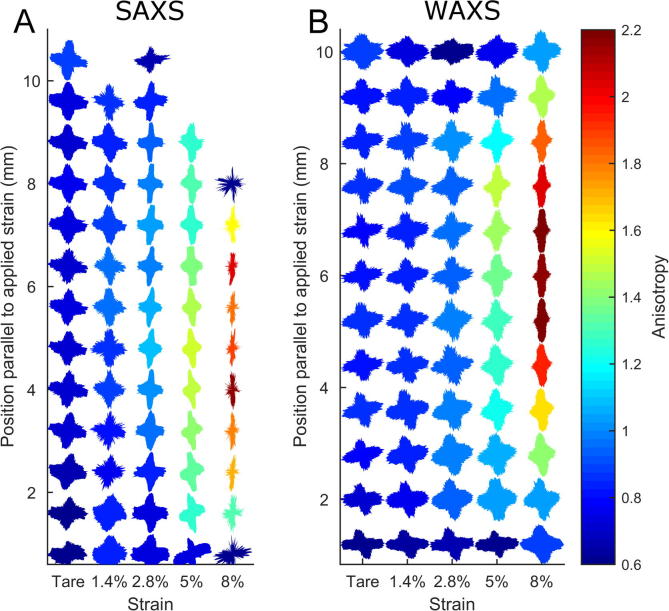
Representative polar patch plots of collagen fibril orientation calculated from the interfibrillar peak distribution observed using SAXS (A) and the intermolecular peak distribution observed using WAXS (B), for representative specimens at varying strain. The anisotropy value is the ratio of maximum parallel collagen to maximum orthogonal collagen. Some SAXS plots are noisy or omitted due to loss in signal associated with disorder at the fibrillar hierarchy.

An example of the normalised polar arrangement of collagen fibrils along the length of a representative specimen, and the variation associated with strain, is shown in Fig. 5A. The equivalent plot for molecular orientation is shown in Fig. 5B. For display purposes each polar plot has been normalised against the signal in the direction of most aligned collagen. As a consequence, the area of each polar plot is of no significance. Both fibrillar and molecular reorientation was evident from the smallest strain increment, although the most pronounced changes occurred between the 2.8% and 5% strain increments. At 8% applied strain the interfibrillar peak became much weaker and broader, making the fibril orientation plot more sensitive to noise. At all strains, the molecular distributions were more isotropic than their fibrillar counterparts, supporting the hypothesis that molecules are tilted relative to the fibril axis.

### 3.2 Local strain effects

**Fig. 6:**
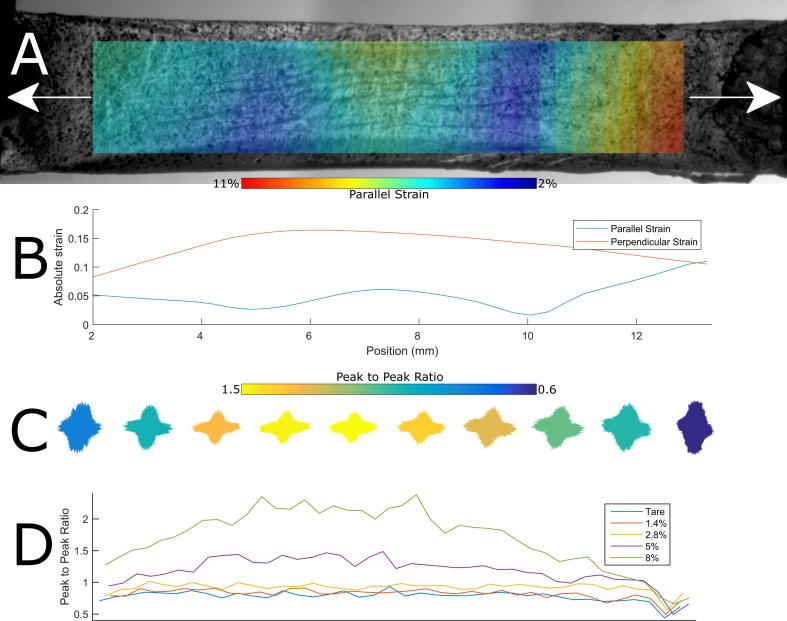
Distribution of local strain and associated reorientation for a representative tensile strip. x-axes of plots coincide. A. Original image of the tensile strip at 5% applied strain with local longitudinal strain field obtained using DIC overlaid. The strain field data are binned into pixels 300 µm in size. B. Average parallel and perpendicular strain along the central longitudinal/parallel axis of the strip, calculated from the central two rows of DIC pixels. C. Polar patch plots of local collagen molecule orientation at 5% applied strain. D. Anisotropy measured as ratio of peak longitudinal to peak orthogonal orientation, for each strain increment.

The distribution of tensile strain was found to be non-uniform along the length of each specimen. Although maximal deformation occurred in the peripheral cornea, the central ∼2 mm of the cornea was found to exhibit greater deformation than the adjacent paracentral region. Fig. 6A shows the local strain field in a representative specimen at 5% applied strain, with the corresponding distributions of absolute longitudinal and orthogonal strain along the widthways centre of the strip shown in Fig. 6B. The perpendicular strain was between 2 and 3 times that in the longitudinal direction, and is clearly non-uniform as evidenced by the formation of longitudinal creases. The variation in anisotropy at high strains is similar to the distribution of perpendicular strain, and it is for this reason that the statistics shown in Fig. 4 are calculated from the lengthways central 2 mm of each sample. The anisotropic arrangement of collagen molecules at 5% applied strain is shown in Fig. 6C, and the variation in anisotropy (defined as ratio of peak parallel signal to peak perpendicular signal) with length at all strains is shown in Fig. 6D. This shows that reorientation as a mechanism for distributing local strain only becomes significant above 2.8% applied strain, and therefore at smaller strains another mechanism must dominate. The pattern of reorientation with strain correlated with the distribution of orthogonal strain and approximately with distance from the fixed ends, although in all specimens and all strains the central 3–4 mm showed even extents of reorientation.

### 3.3 Scatter model

**Fig. 7:**
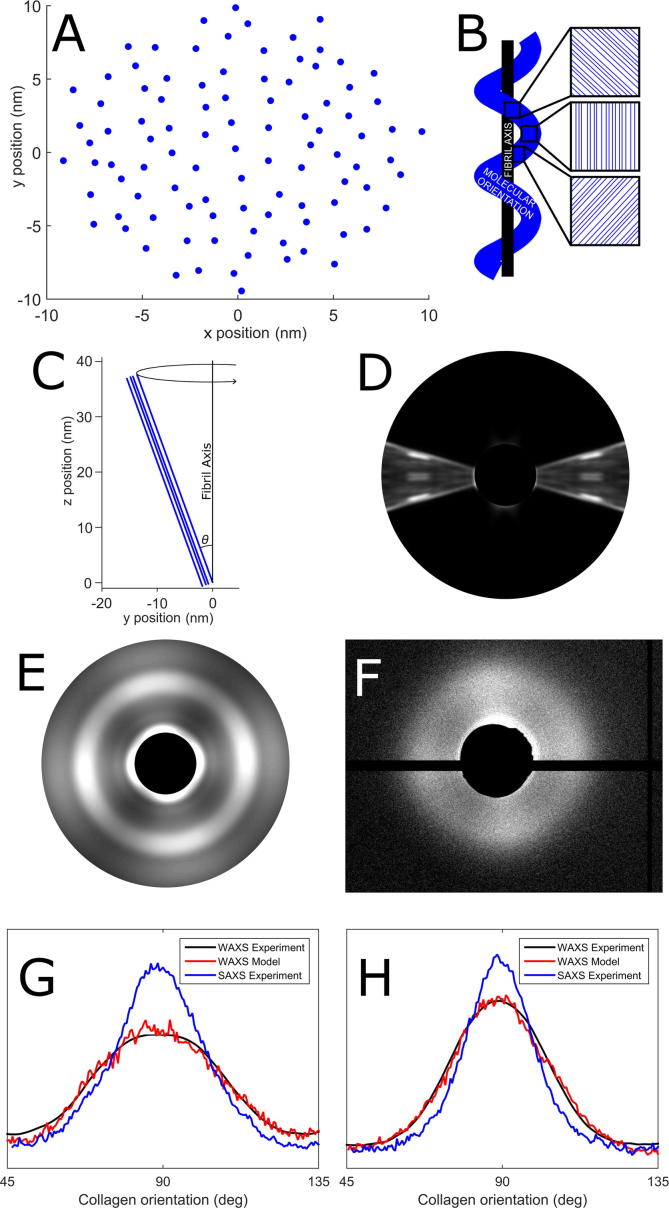
Molecular model geometry and results. A. Axial view of representative line/filament arrangement, which is a disordered hexagonal lattice. B. Illustration of how a helical molecular arrangement (blue) relative to the fibril axis (black) is modelled by a sum of straight line geometries of varying inclination. C. Side view of line model geometry, with molecular tilt shown as inclination angle *θ*. For each value of *θ*, 180 scatter patterns were calculated at 2° azimuthal increments to simulate an arrangement of parallel helices. D. Scatter pattern from the disordered helix model with a tilt of 16°. E. Modelled scatter pattern from a helical model with tilt 16°, convoluted with the average fibril distribution at the tare load. F. Representative WAXS pattern from a specimen under the tare load. G. Averaged polar arrangement of fibrils (blue), molecules (red) and modelled molecules at a 16° tilt (black) for a specimen under the tare load. F. Polar arrangements for a specimen at 5% applied strain, with a fitted 11° tilt.

WAXS scatter patterns are a convolution of the fibrillar orientation and the molecular tilt relative to the fibril axis (Fig. 1). The disordered hexagonal lattice arrangement of molecules is shown in Fig. 7A. Assuming that tropocollagen molecules can be represented as helices with axes parallel to their respective fibril, the sum of several line models of fixed inclination and varying azimuth can simulate the helical arrangement of collagen molecules in a single fibril, as shown in Fig. 7B and C. A modelled WAXS pattern for a single fibril with a molecular tilt of 16° is shown in Fig. 7D. The convolution of this modelled WAXS pattern with experimental fibril orientation data (obtained using SAXS), produced the modelled WAXS pattern showed in Fig. 7E. The distribution of X-ray scatter intensity in the modelled WAXS pattern (Fig. 7E) is in close accord with the experimentally obtained WAXS pattern from the cornea at the tare load (Fig. 7F).

Using the average experimentally measured polar distribution of molecules aligned in the parallel direction range as a fitting parameter, it was possible to use the model to find the corresponding average molecular tilt at each strain increment. Fig. 7G and H show averaged experimentally obtained fibrillar and molecular orientation distributions and the corresponding molecular model at the tare load and 5%, respectively. To a tolerance of 1°, the average tilts are shown in Table 1. The significant drop in molecular tilt may be indicative of a subfibrillar strain mechanism, in that the molecules appear to be stretching like coiled springs. It was not possible to use this approach to calculate tilt at 8% applied strain due to irregularities in the polar arrangement of both fibrils and molecules.

**Table 1:** Molecular tilt at varying strain.

Applied Strain	Average molecular tilt with respect to fibril axis
Tare	16°
1.4%	14°
2.8%	12°
5%	11°

## 4 Discussion

The molecular and fibrillar architecture of corneal collagen has been examined under tensile load. The fibrillar arrangement in the tare loaded state revealed preferential orientation in the superior-inferior and nasal-temporal directions in the central and paracentral cornea, changing to a circumferential preference through the periphery and into the limbus, which is in line with previous reports of unloaded human cornea with an intact scleral rim [30]. Straining the tissue by as little as 1.4% perturbed the arrangement at both the molecular and fibrillar scale. The most pronounced change was in the crystallinity of the fibril network (a drop of 21% ± 3% overall at 5% applied strain), which probably explains the increased opacity of the tissue. While the interfibrillar spacing was found to vary considerably under strain, the average value did not change significantly (increasing by 3.0% ± 1.7% at 5% applied strain), with the results indicating a weak positive trend. This positive trend suggests auxetic behaviour (negative Poisson’s ratio, here meaning a tensile force gives rise to a lateral expansion) at the fibrillar scale, which has been reported in cornea previously at much higher strain and attributed to the proteoglycan network maintaining a minimum interfibrillar distance [42]. At the macroscopic scale, DIC measurements showed that the specimen behaved like a typical biological material, with a high Poisson’s ratio (greater than 1, meaning the magnitude of perpendicular strain is greater than the parallel strain). The inconsistency between behaviours at the fibrillar and macroscopic scale is associated with the formation of longitudinal creases, which presumably took up all the orthogonal macroscopic strain. High Poisson’s ratios are common in soft tissue in tension, and have been reported, for instance, in articular cartilage [43]. The trend in intermolecular strain is similar to that of the fibril diameter, as would be expected, although the slightly larger strain in fibril diameter (3.4% ± 1.3% vs 2.2% ± 1.1%) may indicate shrinkage in the proteoglycan coating surrounding the fibrils [44]. Comparison of volumetric changes at the molecular and fibrillar scale suggests a small load-induced flow of water from the intrafibrillar to the interfibrillar compartment.

**Fig. 8:**
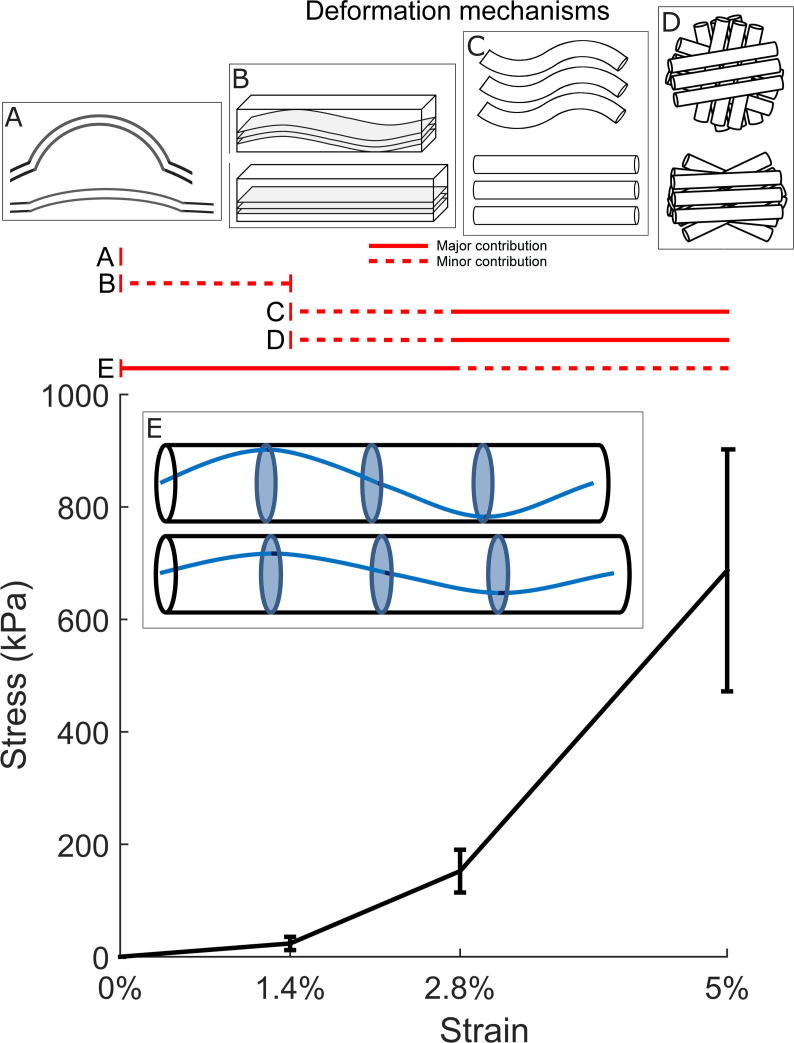
Average quasi-equilibrium tensile stress associated with each strain increment, with spans indicating ranges of strain over which illustrated deformation mechanisms may apply. A. Straightening of corneal curvature, occurs during application of tare load (millimetres). B. Lamellar crimp, arguable contribution at low strains based upon literature evidence [46], [47] (100 s of microns). C. Fibrillar crimp measured using SAXS (100 s of nanometres). D. Lamellar and fibrillar reorientation measured using SAXS (100 s of nanometres). E. Straightening of molecular helical tilt relative to fibril axis measured using SAXS and WAXS (Angstroms).

The applied deformations manifest themselves at several hierarchical scales, which are illustrated in Fig. 8. Firstly the application of the tare load straightens the specimen (Fig. 8A) and applies a small strain differential across the thickness [45]. Lamella inclination angle ranges have been measured using second harmonic generation microscopy following pressure fixation and found to average approximately 12° [46]. While much of the angle is likely to be associated with lamellar interweaving, some may be associated with residual crimp (Fig. 8B). Analysis by Grytz and Meschke on the extensibility of crimped tissue [47] would suggest any residual crimp would be taken up by the first strain increment. The linear trends in fibril strain (from D-period measurements) and changes in molecular tilt measured in this study over the 0% to 2.8% increments would suggest that if any lamella-scale crimp does persist past the tare load, it is not a significant deformation mechanism. Corneal fibrillar crimp (Fig. 8C) has been reported in second harmonic generation [48] and electron microscopy [49] studies, the latter of which showed residual crimp following an applied tensile strain of 10%. In this study the polar arrangement of fibrillar collagen was found to change significantly at the 2.8% strain increment and above, but given that the fibrillar scatter from all lamellae contribute equally to the scatter pattern it is not possible to separate crimp straightening with reorientation of lamellae (Fig. 8D). Evidence of lamella reorientation comes from the changes in the measured ratio of parallel to perpendicular fibril scatter, as without large-scale reorientation fibrils aligned close to perpendicular should not be significantly perturbed by the applied strain. Clearly lamella reorientation is an artefact of extensometry, associated with macroscopic changes in specimen morphology. Preliminary studies not shown in this report found anomalous arrangements of collagen in the region within 600 µm of a cut edge, but outside that region the arrangement was uniform across the width of the specimen. The cut edge of specimens was still well-defined at the end of the experimental protocol, meaning that cut fibril slippage is an unlikely mechanism for lamella reorientation. Homogeneous calculations of predicted fibril reorientation, which involve applying the local deformation obtained via DIC to the fibril orientation distribution at rest, were found to not account fully for the extent of reorientation observed. It is likely, then, that lamella reorientation is caused by a combination of macroscopic geometry changes (the applied strain and the orthogonal contraction), as well as the straightening of intertwined lamellae.

The straightening of molecular tilt relative to the fibril axis (Fig. 8E) in a manner akin to a spring, has not been quantified before, but atomistic modelling has suggested it may be an important deformation mechanism at small applied loads [50]. It has been suggested that collagen fibrils fall into two types: [51] the T-type (found for example in tendon) being heterogeneous in diameter, with a small molecular tilt (<5°) and exhibiting significant crimp when unloaded; and the C-type, native to the cornea and other tissues such as cartilage and skin, which are uniform in diameter and molecular tilt (17°), and are significantly more flexible than T-type fibrils [52]. The calculated tilt of 16° for tare loaded specimens supports the hypothesis that the spread of molecular orientation about the fibrillar arrangement is due to the helical tilt of molecules. Continuing with the helical approximation, the observed changes in molecular tilt at strain increments (1.4%, 2.8%, 5%) would suggest the fibrils are deforming by an average of (0.9%, 1.8%, 2.1%). Given that the tilts were calculated for fibrils oriented within a range ±45° about the direction of applied strain, it is possible that the significant majority of the deformation in parallel-aligned collagen is taken up by molecular straightening up to the 2.8% increment, with other mechanisms being invoked beyond that. The dominance of this “spring-like” deformation mechanism at low strains suggests it may be a fundamental regulator of the mechanical response of the cornea to changes in IOP in vivo, as well as the mechanical response of other tissues rich in C-type collagen that experience small physiological strains. A problem with this interpretation is that the D-period is intrinsically linked with molecular tilt *α* via the relation $\cos\alpha =D_{c}/D$ where *D_c_* is the measured corneal D-period and *D* is the molecular stagger [53]. The measurements of D-period in this study do not obey this relation, as at 5% applied strain, and 11° tilt the measured D-period change in the parallel direction should be 2.1% rather than 0.6%. This discrepancy may be an artefact of using meridional peak positions in the calculation of the D-period rather than spread or range, as it was noted qualitatively that the peaks change shape as strain increased.

In accordance with previous work involving inflation testing, [18], [27], the peripheral cornea was found to deform more than the medial regions. It is possible that such a mechanism prevents changes in intraocular pressure from significantly affecting focus. The recent discovery of an elastic sheet-like network in the periphery [31], [54] has strengthened suggestions that the peripheral cornea acts as a strain absorber, deforming under changes in intraocular pressure and thereby minimising changes in curvature in the paracentral and central cornea [29], which could be detrimental to vision. Such a system would function best if the deformation was as efficient as possible, and a synergy between networks of near-lossless elastic fibres and collagen fibrils that can deform longitudinally via a subfibrillar mechanism may be the optimal arrangement. Longitudinal fibril deformation will also limit changes in lateral packing that could have an adverse effect on corneal transparency. The increased strain in the central cornea compared with the paracentral region has been reported before under inflation testing [34], and was associated with increased membrane stress (due to variations in curvature). It is possible that geometry also explains this result, as under the tare load orthogonal creases were evident in the central cornea, which were presumably due to the straightening of a normally curved structure [45], giving rise to internal stress. The fact that the D-period drops by a significant amount (decreasing from 65.1 nm to 64.9 nm) in the orthogonal direction under load would suggest the collagen network is under significant internal stress in vivo which is likely to be associated with the swelling pressure of the hydrated glycosaminoglycan network. This phenomenon has been investigated recently in articular cartilage [55], where the internal stress is likely to be significantly higher. The orthogonal D-period does not change significantly beyond the initial 1.4% strain increment, which corresponds to an average orthogonal strain of 1.1%. Assuming the decreased D-period is derived from collagen released of all internal stress, corneal internal pressure could be derived from consideration of the stress-strain behaviour of corneal collagen fibrils.

Keratoconus is regarded as a degenerative disease affecting the corneal collagen network, in which a degeneration of the fibrillar structure and an increased propensity of fibrils to slide relative to one-another gives rise to altered macroscopic morphology and astigmatism [16]. It is possible that in this study some fibril sliding occurred at the higher strain increments, as the changes in the polar distribution of parallel fibrils (reflecting straightening of crimp or reorientation of lamellae), combined with the elongation of fibrils measured via molecular tilt calculations, does not account for the magnitude of the applied strain. To determine the magnitude of sliding, an approach that incorporates light microscopy, such as previous studies on tendon [8] and intervertebral disc annulus [56] could be utilized. It is likely that a combined approach of mechanical stimulus and X-ray scattering, such as that presented here, on keratoconus corneas could yield valuable information about changes in hierarchical mechanical properties of diseased tissue. This approach may also have utility in determining differences in the micromechanical properties of healed/scarred tissue following invasive surgery [57], [58], or tissue treated using crosslinking techniques [59].

Many of the techniques demonstrated in this study are transferable to other tissues. Measurement of the orientation of fibrils is possible by quantifying the azimuthal distribution of signal in the meridional peaks, which are ubiquitous to all collagenous tissue, while the intermolecular spacing peak features in all tissues, and provides a measurement of molecular orientation. Comparison of fibrillar and molecular orientation via the modelling approach shown in this work then provides a measure of molecular tilt relative to the fibril axis. This has the potential to be a powerful tool, as the change in molecular tilt is related to the stress within the fibril. Changes in the tilt response of tissue under known loads in, for instance, degenerative diseases such as osteoarthritis, or fibrosis associated with vascular disease, is indicative of a change in the fibrillar mechanical environment. Such changes could be quantified to enhance the accuracy of models of disease. The time-dependence of this deformation mechanism remains to be elucidated, and a transient study could help further characterise its function. To measure the precise contribution of each mechanism in vivo, a more physiologically-realistic loading regime would need to be adopted, such as bi-axial extensometry, or inflation testing. As a follow-up to this study we plan to carry out stress-controlled inflation testing combined with SAXS/WAXS to most closely mimic the in vivo mechanical environment. The findings presented in this study could be incorporated into the design of collagen-based artificial corneas that are being developed to address world-wide shortages of corneal donor tissue, and other regenerative medicine applications involving connective tissues [60].
